# Romanian male patients with the dual diagnosis of schizophrenia and alcohol use disorder: a prospective study of clinical, social, and treatment-related factors affecting quality of life

**DOI:** 10.3389/fpsyt.2026.1780813

**Published:** 2026-05-13

**Authors:** Antonia Ioana Vasile, Simona Trifu, Cristina Alexandra Negoita

**Affiliations:** 1 Doctoral School, “Carol Davila” University of Medicine and Pharmacy Bucharest, Bucharest, Romania; 2 Department of Neurosciences, “Carol Davila” University of Medicine and Pharmacy Bucharest, Bucharest, Romania

**Keywords:** alcohol use disorder, dual diagnosis, psychosocial factors, quality of life, schizophrenia

## Abstract

**Background:**

Schizophrenia frequently co-occurs with alcohol use disorder (AUD), resulting in a complex clinical profile associated with poor functional outcomes and reduced quality of life (QoL). Although both conditions independently impair psychosocial functioning, few studies have examined the combined effects of clinical, social, and treatment-related factors on QoL in patients with this dual diagnosis.

**Methods:**

This prospective observational study included 88 male inpatients diagnosed with schizophrenia and comorbid AUD and who were followed over a 6-month period. Quality of life was assessed using the World Health Organization Quality of Life–BREF (WHOQoL–BREF). The clinical variables included severity of psychotic symptoms (Positive and Negative Syndrome Scale), alcohol use severity (Michigan Alcohol Screening Test), and treatment characteristics. Social and personal factors, such as self-care capacity, social support, education, and legal problems, were also evaluated. Multivariable regression analyses were conducted to identify predictors of QoL at baseline and follow-up.

**Results:**

At baseline, higher QoL was significantly associated with greater self-care capacity, social support, and higher positive symptom scores, while the need for antipsychotic treatment was associated with lower QoL. At the 6-month follow-up, better QoL was predicted by greater self-care capacity, higher educational level, and receipt of anti-craving medication. By contrast, negative and general psychopathology, medico-legal problems, and the need for antidepressant treatment were associated with poorer QoL. Alcohol use severity, as measured by the MAST, was not independently associated with QoL at either timepoint.

**Conclusions:**

In patients with schizophrenia and comorbid AUD, QoL is shaped by a complex interaction of clinical severity, functional capacity, and treatment-related factors. Beyond symptom control, interventions targeting self-care, social functioning, and integrated addiction treatment appear essential to improve long-term outcomes. These findings support the implementation of a multidimensional, recovery-oriented approach for the management of patients with the dual diagnosis.

## 1 Introduction

Schizophrenia is frequently complicated by hazardous alcohol consumption and alcohol use disorder (AUD), a comorbidity that intensifies psychopathological symptoms, impairs the course of treatment, and negatively impacts quality of life (QoL). Current epidemiological research indicates that substance use disorders (SUDs) occur in roughly a quarter of patients with schizophrenia in population-based cohorts, with clinically meaningful rates of alcohol involvement; in particular, these patterns are more prevalent among men and younger patients, and they are associated with more serious illness progression and increased healthcare burden (1, 2). Co-occurring SUD systematically worsens outcomes compared with those in single-diagnosis groups (higher relapse rate, increased use of emergency services, higher rehospitalization rate, and even higher mortality), highlighting the need to target both conditions as a unitary clinical entity (3).

Over the last decade, QoL has become a primary measure of recovery rather than a secondary outcome in both schizophrenia and dual diagnosis. The results of generic and disease-specific instruments converge to demonstrate that symptom burden, unmet psychosocial needs, and environmental constraints are related to diminished perceived well-being (4, 5). In schizophrenia cohorts, higher positive, negative, and depressive sub-scores on the Positive and Negative Syndrome Scale (PANSS) correlate with reduced health-related quality of life (HRQoL) across multiple measures; negative symptoms and depression, in particular, exert broad, cross-instrument effects (4, 6). Studies that assessed experiential versus expressive negative symptoms suggest that experiential deficits (such as anhedonia or avolition) are especially toxic to subjective QoL, which helps explain the stubborn gap between symptomatic improvement and subjective recovery (6).

Alongside symptom profiles, functional and social determinants have become key factors that influence QoL. Multivariable models repeatedly show that social support, work-life balance, and patient-reported needs predict QoL beyond symptom severity, implying that recovery capital (personal skills, social supports, and tangible resources that support recovery) mediates the transition from clinical stabilization to well-being (7, 8). Family ecosystems matter as well; specifically, caregiver burden and professional support influence family QoL and patient outcomes, highlighting the need for family-focused and community-based interventions in dual diagnosis care (9).

The measurement of QoL remains a methodological pillar. The World Health Organization Quality of Life–BREF (WHOQoL–BREF), an instrument used to measure QoL, continues to have strong psychometric support and cross-cultural validity while assessing physical, psychological, social, and environmental domains that are sensitive to treatment and context (10). Meanwhile, schizophrenia-specific scales (e.g., SQLS-R4) have also been refined and validated in diverse settings, improving the domain coverage for negative symptoms, cognitive–affective features, and antipsychotic side effects (11, 12). For assessing psychopathological symptoms, the PANSS remains a gold-standard scale, and its results can be robustly mapped onto preference-based HRQoL utilities, facilitating health–economic evaluation (13, 14).

Alcohol use exacerbates psychosis through multiple mechanisms. From a neurobehavioral viewpoint, intoxication, withdrawal, and alcohol-related cognitive deficits amplify disorganization, worsen insight, and destabilize sleep and mood, all of which decrease QoL (15, 16). Epidemiological studies in community and clinical samples have consistently reported higher rates of AUD among patients with serious mental illness compared to among the general population, with associated impairments in functioning, housing stability, and social integration (17, 18). In schizophrenia cohorts, hazardous drinking correlates with poorer functioning and lower QoL, independent of symptom severity, underlining alcohol’s distinct role in increasing the disability burden (7).

Against this background, there is a critical need for reliable screening tools for alcohol problems. While numerous brief tools exist, the Michigan Alcohol Screening Test (MAST) remains one of the most widely used lifetime problem-drinking screening tools in psychiatry and addiction services. When combined with structured clinical assessment, the MAST facilitates both severity staging and treatment monitoring. Recent comprehensive syntheses have emphasized naltrexone/acamprosate as first-line treatment for AUD and have recommended integrated measurement-based care for severe mental illness (SMI), in which validated alcohol measures are incorporated into psychiatric follow-up (19, 20).

Expert consensus and emerging evidence support the use of integrated dual diagnosis treatment (IDDT) over that of parallel or sequential care. IDDT programs combine motivational interviewing, cognitive behavioral therapy (CBT)-based relapse prevention, case management, and coordinated pharmacotherapy; such programs are feasible, acceptable, and associated with improvements in substance outcomes and functioning, although effect sizes vary across settings (21, 22). Long-term integrated-care studies have confirmed improvements in patients with SUD, but psychopathological symptoms, general functioning, and QoL in these patients remain worse than those in non-SUD peers, suggesting persistent obstacles even when evidence-based care models are implemented (23).

From a pharmacological perspective, the anti-craving agent, naltrexone, has accumulated convergent evidence supporting its safety and efficacy in reducing alcohol consumption in patients with schizophrenia and comorbid AUD, particularly when adherence is actively supported; however, the body of evidence is still small (24, 25). New reviews of SMI plus SUD pharmacotherapy highlight naltrexone as the most consistently effective AUD medication in these patients while stressing the importance of early and intensive psychosocial interventions, alongside judicious selection of antipsychotics (choosing antipsychotics with fewer side effects or depot antipsychotics to increase adherence) (20). Not all pharmacological innovations demonstrate superior efficacy—for example, olanzapine/samidorphan was not superior to olanzapine in preventing symptom exacerbations or reducing the number of heavy drinking days in patients with schizophrenia and AUD; this reminds clinicians that progress occurs gradually, and individualized treatment remains crucial (26). Overall, recent syntheses converge on the need for personalized, measurement-based pharmacotherapy combined with integrated psychosocial care for dual diagnosis treatment (27).

Despite this growing body of literature, key knowledge gaps persist. First, relatively few studies have analyzed QoL as a dependent variable in dual diagnosis while simultaneously testing multidomain predictors, such as psychotic symptoms (PANSS domains), alcohol severity, medication variables (antipsychotics, antidepressants, anti-craving agents), hospitalization history, and social determinants (self-care, social support, financial planning, education). Existing research reveals that both symptoms and social context influence QoL, yet integrated models that assess their changing contributions throughout the treatment period remain limited (28, 29). Second, although social support consistently emerges as a protective factor for recovery and QoL, its interaction with pharmacological choices (e.g., anti-craving therapy) remains underexplored in schizophrenia comorbid with AUD (8, 9, 30). Third, many QoL studies are cross-sectional; longitudinal studies that track how self-care capacity, education, and medication choices predict QoL at 6 months into care are needed to guide service planning and personalized interventions (23).

Given the abovementioned gaps, the aims of this study were as follows. First, the present research aimed to evaluate the difference in QoL among patients with schizophrenia and comorbid alcoholism during a 6-month follow-up period based on multidimensional factors. To this end, we performed two regression analyses, with QoL as the dependent variable (first analysis at baseline and second analysis at the 6-month follow-up) and multiple factors as independent variables.

Second, the study aimed to identify the factors that influence QoL in patients with the dual diagnosis and to determine how these factors differ between baseline and follow-up.

The research hypotheses addressed how several dimensions of the psychopathology of patients with the dual diagnosis can positively or negatively affect QoL. The first research hypothesis was that alcohol severity would negatively impact QoL and that using anti-craving medication would increase QoL. The second research hypothesis was that the general psychopathology and negative dimensions of schizophrenia would negatively influence QoL and that adding antidepressant medication to antipsychotic medication would increase QoL. The third research hypothesis was that a higher level of social functioning would protect the patient, leading to improved QoL. The fourth research hypothesis was that certain individual factors (e.g., age, educational level, or area of residence) would act as protective factors for the QoL of patients with the dual diagnosis.

Although these factors have been extensively examined in previous studies, the novelty of the present study stems from two key elements. First, we employed a multidimensional approach to patient assessment, encompassing multiple factors related to AUD, schizophrenia, social functioning, individual patient’s personal lives, hospitalization, and treatment. Second, we evaluated the effects of anti-craving treatment on the clinical course of patients with the dual diagnosis over a 6-month follow-up period.

## 2 Materials and methods

### 2.1 Study design

This study employed a prospective, non-randomized observational design, in accordance with the CONSORT extension for non-randomized evaluations of interventions. All participants were assessed at baseline and at 6-month follow-up.

Treatment decisions were made by the attending psychiatrist based on clinical indications, including symptom severity and standard-of-care considerations. Consequently, pharmacological management was tailored to each patient and typically included combinations of antipsychotic, antidepressant, and, where appropriate, anti-craving medication. Randomization was not feasible due to the need for individualized treatment.

To mitigate potential bias associated with this design, standardized assessments were conducted at both timepoints (baseline and 6-month follow-up), and multivariable analyses were used to adjust for relevant demographic and clinical factors.

Recruitment was concluded once a balanced sample was obtained, which comprised 45 patients receiving anti-craving medication and 43 not receiving such treatment. This approach was guided by one of the study’s research objectives, namely, to examine whether the use of anti-craving medication is associated with differences in QoL among patients with dual diagnosis.

### 2.2 Participants

A total of 110 patients were assessed for eligibility, of whom 88 met the inclusion criteria and were enrolled in the study.

The enrolled patients were adult inpatients (aged 18 years or older) from a male psychiatric ward of the main clinical psychiatry hospital in Bucharest, Romania. According to the DSM-4 diagnostic standards, the primary Axis I diagnosis of the included patients was the dual diagnosis of schizophrenia and alcohol dependence. However, according to the DSM-5 and ICD-10 diagnostic standards, all participants were initially diagnosed with schizophrenia and subsequently developed alcohol abuse or dependence, resulting in AUD. Their ages ranged from 23 to 80 years (mean = 47.20, standard deviation [SD] = 13.15). We enrolled patients who were admitted both through voluntary (45 patients, 51.13%) and involuntary (43 patients, 48.86%) hospitalization.

### 2.3 Inclusion criteria

The first inclusion criterion was the confirmed diagnosis of schizophrenia, along with the confirmed diagnosis of AUD. Both diagnoses were established based on an unstructured clinical assessment conducted by a psychiatrist according to DSM-5 and ICD-10 criteria. The second inclusion criterion was a history of at least two prior psychiatric hospitalizations, either at the site of the present study or at other psychiatric institutions. For this requirement, one previous hospitalization should have been for schizophrenia-related episodes, while the other previous hospitalization, for alcohol-related episodes. The third inclusion criterion was a primary admission for a complication of AUD rather than for an acute psychotic relapse of schizophrenia. The final inclusion criterion was adherence to treatment and compliance with medical recommendations, ensuring participation in the 6-month follow-up assessment.

### 2.4 Exclusion criteria

Patients were excluded if they had severe psychiatric comorbidities, such as major affective disorders, psychotic disorders other than schizophrenia (e.g., schizoaffective disorder), or severe personality disorders that could affect assessment (e.g., borderline or antisocial personality disorder).

Patients were also excluded if they had severe organic comorbidities requiring treatment in specialized units (e.g., hepatic encephalopathy, severe alcoholic polyneuropathy, liver cirrhosis, liver failure, chronic hepatitis B or C, cardiomyopathy, or heart failure). Patients with neurological conditions (such as degenerative neurocognitive impairment or borderline intellectual functioning) were also excluded. Patients with active substance abuse other than alcohol abuse were also excluded. Additionally, patients with autoimmune or infectious diseases (including HIV) and non-compliant patients were also excluded.

### 2.5 Assessment procedure

At admission, either voluntary or involuntary, 42 patients (47.72%) presented with uncomplicated or complicated withdrawal symptoms, including delirium tremens. For these patients, psychometric evaluations were conducted between days 7 and 12 of hospitalization once they had regained clear consciousness and were able to actively participate in interviews and complete the questionnaires. For the other 46 patients (52.27%), psychometric evaluations were conducted between days 1 and 3 of hospitalization.

**Figure 1:**
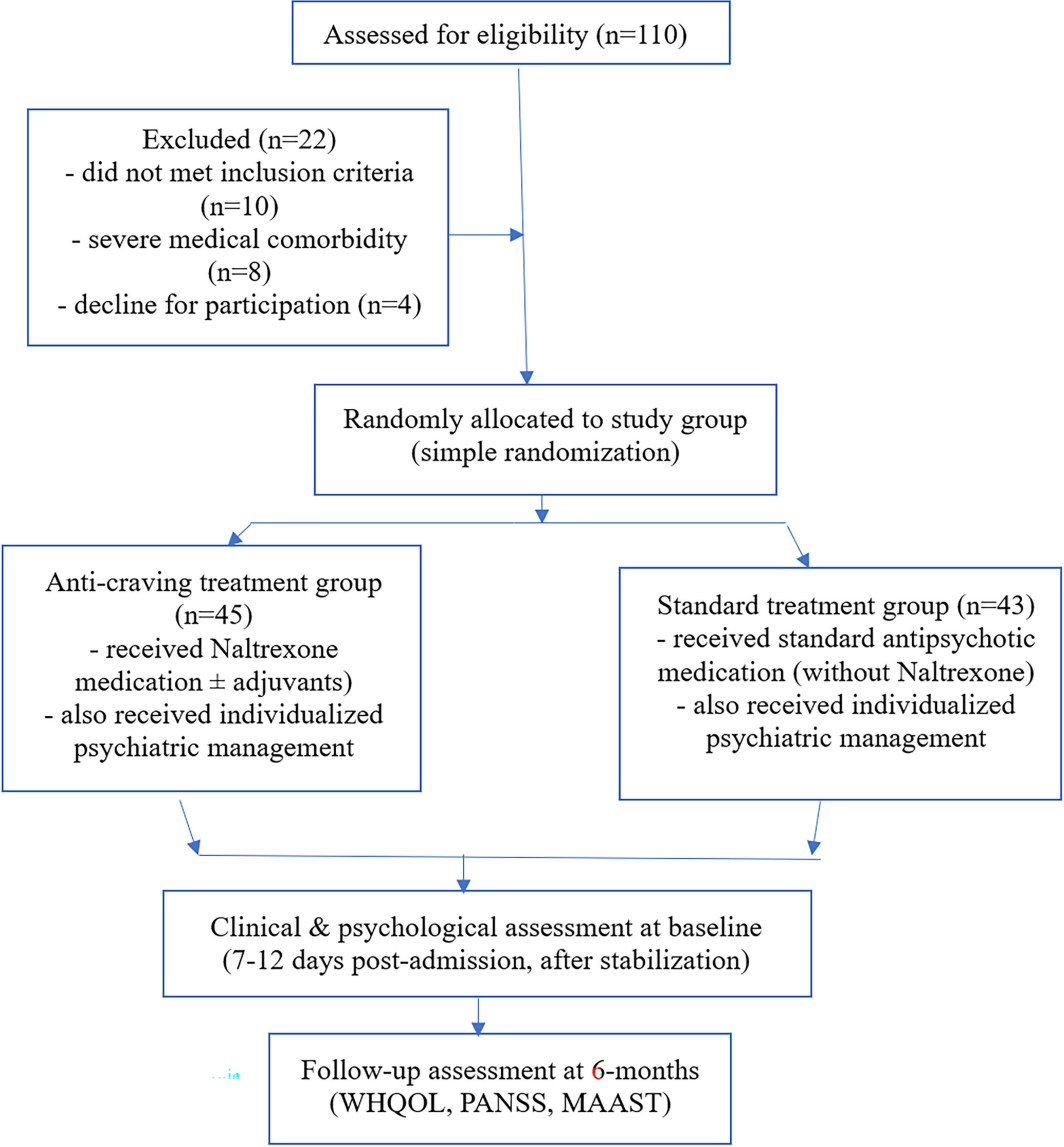
Flow of participants through the study (CONSORT-style diagram).

Assessments were conducted in both the physician’s and department psychologist’s offices. Hospital stays ranged from 7 to 21 days. The flow of participants is presented in **Figure 1**.

### 2.6 Instruments and outcome measures

Each participant was evaluated from a multidimensional perspective by taking the following seven categories of factors (or dimensions) into account: QoL, AUD severity, schizophrenia severity, social factors, personal factors, hospitalization factors, and treatment factors.

The instruments and outcome measures were selected based on previous research in the field. The instruments and outcome measures are as follows:

1. QoL variable: QoL was measured as a numeric variable using the WHOQoL–BREF, which comprises 26 items rated on a Likert scale of 1 to 5, with higher scores reflecting better QoL.

2. AUD severity: This numeric variable was measured using the MAST, which comprises 25 items, each scored dichotomously (“yes/no”). The total score is obtained by summing the item responses, with higher scores indicating greater severity of AUD.

3. Schizophrenia severity: This was measured using the PANSS, which is composed of three subscales:

- Positive scale (numeric variable): This subscale comprises seven items rated on a Likert scale of 1 to 7: delusions, conceptual disorganization, hallucinations, excitement, grandiosity, suspiciousness, and hostility.
- Negative scale (numeric variable): This subscale comprises seven items rated on a Likert scale of 1 to 7: blunt affect, emotional withdrawal, poor rapport, passive/apathetic social withdrawal, difficulty in abstract thinking, lack of spontaneity and flow of conversation, and stereo-typed thinking.
- General psychopathology scale (numeric variable): This subscale comprises 16 items rated on a Likert scale of 1 to 7: somatic concern, anxiety, guilt feelings, tension, mannerisms and posturing, depression, motor retardation, uncooperativeness, unusual thought content, disorientation, poor attention, lack of judgment and insight, disturbance of volition, poor impulse control, preoccupation, and active social avoidance.

The PANSS was administered to all patients both at the initial and final timepoints by the same rater accredited in using this scale, thereby ensuring that the related questions were used accurately. In this context, backed by extensive clinical experience, the rater was able to correctly differentiate whether the positive score on the PANSS scale was related to the productive symptoms of schizophrenia and not to psychosis caused by alcohol consumption. Somatic impairment caused by fatigue, asthenia, and subjective malaise is scored on the PANSS general symptomatology scale, and the resulting score obviously represents a cumulation of the psychological discomfort caused by hallucinations, delusions, and the negative side effects of alcohol.

4. Social factors: These factors were assessed as dichotomous variables; specifically, the researchers recorded whether the following factors were present using “yes/no” responses: social support, presence of social stressors, self-care capacity, presence of medico-legal complications, risk to public safety, employment stability, and financial planning.

5. Personal factors:

- Age: Age was measured as a numeric variable.
- Area of residence: A dichotomous variable specifying whether the patient resided in an urban or rural area was used.
- Level of education: This was measured as a categorical variable based on which the participants were classified into five groups according to educational attainment: (1) middle school, (2) vocational training, (3) high school, (4) university degree, and (5) postgraduate education.

6. Hospitalization factors:

- Type of admission: This was assessed as a dichotomous variable specifying whether the patient was admitted voluntarily or involuntarily.
- Number of previous hospitalizations: This was assessed as a dichotomous variable based on which the participants were divided into two groups, namely:

- A group comprising patients during their third hospitalization (considering that the previous two hospitalizations were hospitalizations in which the patients received the dual diagnosis for inclusion in the present research).
- A group comprising patients with more than three hospitalizations.

7. Treatment factors: This categorical variable indicated whether each patient received antidepressant (we used trazodone), antipsychotic (we used amisulpride), or anti-craving treatment (we used naltrexone).

The antidepressant, trazodone, was administered as follows: during the first 3 days, the patients received 75 mg, and from the fourth day, they received 150 mg. We chose trazodone because it is considered efficient at this dosage, without exerting a sedative effect: none of the patients experienced sedation-type reactions—specifically, reactions that would produce a difference in the patients from the time of receiving this medication to the following days. Some patients received antidepressant treatment during the study period based on clinical judgment, as follows: to regulate insomnia, given that trazodone is a good sleep inducer, or to reduce craving when the treating physician noted multiple relapses in the dimension of alcoholism, considering that the naltrexone–antidepressant combination is more effective than the exclusive use of naltrexone. At the same time, the antidepressant was administered to treat the negative symptoms associated with schizophrenia.

We administered amisulpride as the antipsychotic at a dosage of 400 to 800 mg per day.

The administration of each treatment type (antidepressant, antipsychotic, or anti-craving) was determined by the treating physician based on the individual clinical presentation of each patient in accordance with the principles of personalized therapy.

**Figure 2:**
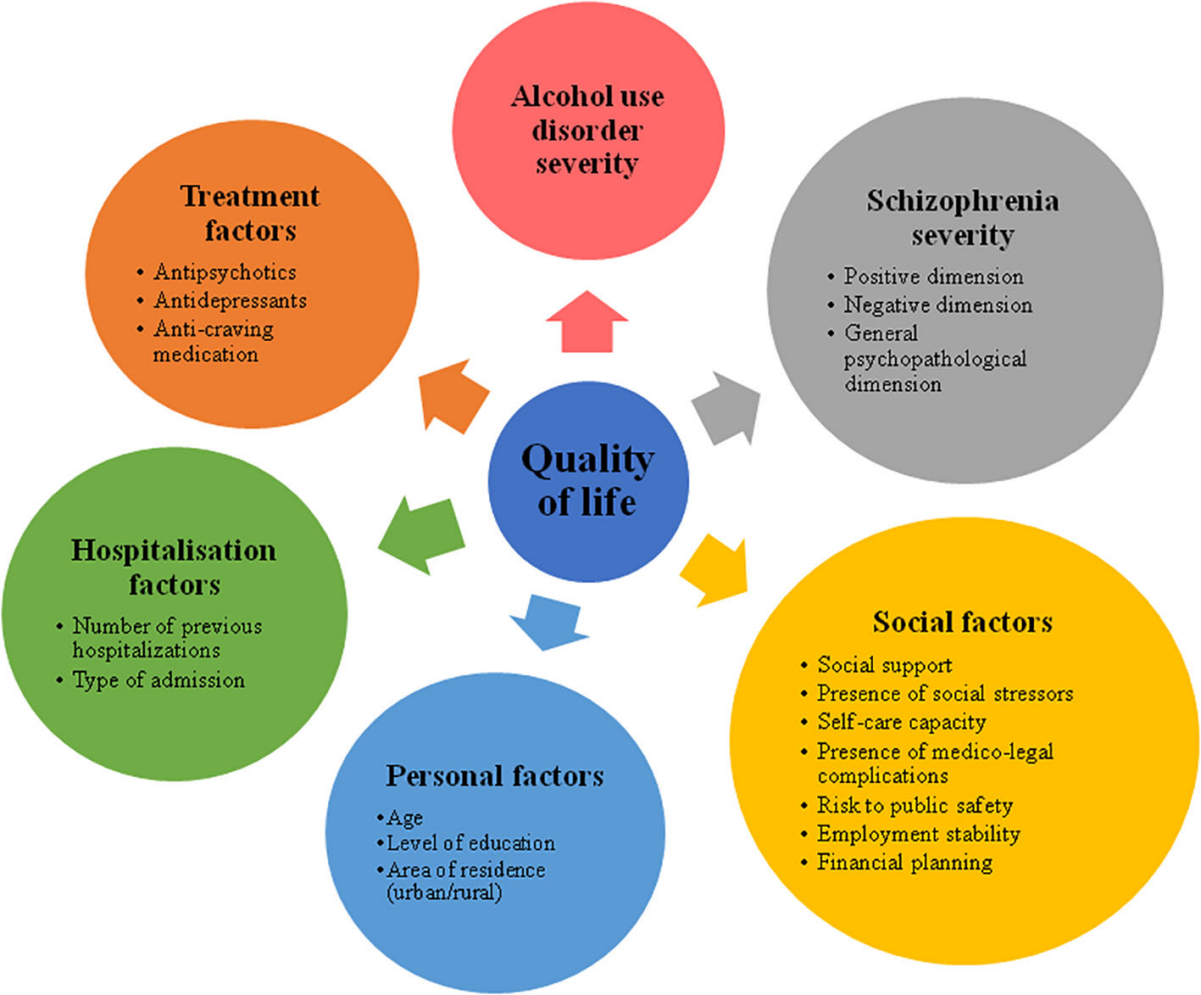
Multidimensional factors assessed in patients with the dual diagnosis of schizophrenia and alcoholism.

The multidimensional factors assessed in our research are presented in **Figure 2**.

### 2.7 Statistical and power analyses

Descriptive statistics were first computed to summarize the demographic and clinical characteristics of the sample. Independent sample *t*-tests were used to compare mean differences between groups, where appropriate. All statistical analyses were performed using IBM SPSS Statistics, with the level of statistical significance set at *p* < 0.05.

To examine the factors associated with QoL (WHOQOL–BREF), multiple linear regression analyses were conducted at two timepoints: baseline and 6-month follow-up. A total of 20 predictors were initially entered in each model, including six continuous variables and 14 dichotomous or categorical variables. A backward elimination method was applied to identify the most relevant predictors of the dependent variable: QoL.

An *a priori* power analysis was conducted to evaluate the adequacy of the sample size for these regression models. On assuming 20 predictors, an alpha level of 0.05, and a desired statistical power of 0.80, the minimum detectable effect size was estimated at approximately *f*² = 0.295, corresponding to an *R*² value of approximately 0.23. Under these assumptions, the available sample size (*N* = 88) was sufficient to detect relatively large overall effects, while smaller or moderate associations may have remained undetected.

## 3 Results

### 3.1 Descriptive statistics

The research sample consisted of 88 male participants.

Regarding personal factors, their ages ranged from 23 to 80 years (mean = 47.20, SD = 13.15). Educational backgrounds were distributed as follows: middle school education, 11 patients (12.5%); vocational training, 16 patients (18.2%); high school education, 31 patients (35.2%); university degree, 24 patients (27.3%); and postgraduate education, six patients (6.8%). Regarding the area of residence, 61 patients (69.3%) were from urban areas, and 27 patients (30.7%) were from rural environments.

Regarding hospitalization factors, 24 patients (27.3%) were experiencing their third hospitalization (given that the dual diagnosis was established during the two previous hospitalizations), while 64 patients (72.7%) had more than three previous hospitalizations. Regarding the type of admission, 46 patients (52.3%) were admitted voluntarily, while 42 (47.7%) were admitted involuntarily.

Regarding treatment factors, the distribution was as follows: At baseline, 76 patients (86.36%) needed an antipsychotic (and 12 patients did not), while at the 6-month follow-up 75 patients (85.23%) still needed an antipsychotic (and 13 patients did not). At baseline, 62 patients (70.45%) needed an antidepressant (and 26 patients did not), while at the 6-month follow-up 46 patients (52.27%) still needed an antidepressant (and 42 patients did not). In terms of anti-craving treatment, we selectively allocated 45 patients to receive anti-craving medication and 43 to not receive anti-craving medication.

Descriptive statistics on analyzing the social factors are presented in **Table 1**.

**Table 1:** Descriptive statistics of social factors.

Social factor	Moment of assessment	No. of patients	%
social support	baseline	58	65.91
	follow-up	62	70.45
presence of social stressors	baseline	51	57.95
	follow-up	41	46.59
self-care capacity	baseline	58	65.91
	follow-up	76	86.36
presence of medico-legal complications	baseline	16	18.18
	follow-up	10	11.36
risk to public safety	baseline	28	31.82
	follow-up	9	10.23
employment stability	baseline	29	32.95
	follow-up	50	56.82
financial planning	baseline	28	31.82
	follow-up	25	28.41

Descriptive statistics on analyzing the numeric variables are presented in **Table 2**; specifically, the results for the scales—the WHOQoL–BREF, MAST, and PANSS scales—divided by the two evaluation timepoints are presented (baseline and 6-month follow-up).

**Table 2:** Descriptive statistics of numerical variables (WHOQoL-BREF, MAST, and PANSS scores). WHOQoL-BREF, World Health Organization Quality of Life–BREF; MAST, Michigan Alcohol Screening Test; PANSS, Positive and Negative Syndrome Scale.

	WHOQoL-BREF		MAST		PANSS positive		PANSS negative		PANSS general	
	baseline	follow-up	baseline	follow-up	baseline	follow-up	baseline	follow-up	baseline	follow-up
Mean	65.52	83.40	44.68	33.67	27.97	20.94	34.70	27.40	69.24	53.77
SD	22.13	25.46	12.23	13.59	10.15	9.57	8.12	9.80	18.48	19.83
Minimum	20	24	16	8	9	9	20	10	18	16
Maximum	110	125	88	79	48	56	46	53	110	99
CI (95.0%)	4.69	5.40	2.59	2.88	2.15	2.03	1.72	2.08	3.92	4.20

### 3.2 Comparison of means using the *t*-test

One of our research hypotheses was that social support would influence QoL; therefore, we prospectively analyzed whether there would be statistically significant differences between the group of patients who received social support and the group of those who did not. To test our hypothesis, we used the independent sample *t*-test (**Table 3**).

**Table 3:** Independent samples *t*-test values for quality of life based on social support.

Moment of testing QoL	Levene test				T test				
	F	p	t	df	Sig. (2 tailed)	Mean Difference	SE of Difference	Confidence interval (95%)	
								Inferior	Superior
WHOQoL-BREF at the baseline	.142	.708	-2.24	86	.028	-11.08	4.93	-20.93	-1.24
WHOQoL-BREF at the follow-up	.993	.322	-3.35	86	.001	-19.33	5.76	-30.79	-7.87

At baseline, the condition of homogeneity of variances was satisfied, with the Levene test producing the following results: *F*(1,86) = 0.142 and *p* = 0.708. Based on the *t*-test results, there was a significant difference between patients who received social support (*M* = 69.33, SD = 26.63) and those who did not (*M* = 57.96, SD = 21.45) in terms of QoL at baseline (*t* = 2.24, *p* = 0.028). The effect size of social support on QoL was medium at baseline (*d* = 0.51).

At the follow-up, the condition of homogeneity of variances was satisfied, with the Levene test producing the following results: *F*(1,86) = 0.993 and *p* = 0.322. Based on the *t*-test results, there was a significant difference between patients who received social support (*M* = 88.67, SD = 23.08) and those who did not (*M* = 69.33, SD = 26.63) in terms of QoL (*t* = 3.35, *p* = 0.001) at the 6-month follow-up. The effect size of social support on QoL was large at the follow-up timepoint (*d* = 0.80).

### 3.3 Regression analysis at baseline

One of the research questions that our study aimed to address was which factors influence QoL at baseline. Accordingly, we performed a multiple regression analysis in which the WHOQoL–BREF score was the dependent variable, and all of the analyzed factors were independent variables: alcoholism severity (measured by MAST), schizophrenia severity (measured by PANSS positive, PANSS negative, and PANSS general), social factors (social support, presence of social stressors, self-care capacity, presence of medico-legal complications, risk to public safety, employment stability, and financial planning), personal factors (age, level of education, area of residence), hospitalization factors (number of previous hospitalizations and type of admission), and treatment factors (the need for an antidepressant, antipsychotic, anti-craving medication, or combination of medications).

For the statistical method, we chose the backward analysis, as this method provides correct statistical models after individually testing all possible models by initially including all variables and subsequently eliminating those that are not relevant one by one. Consequently, 15 models were generated.

We relied on the adjusted *R*^2^ value, which indicated the relevance of the model. The higher the value, the better the model; accordingly, model 10 was chosen (adjusted *R*^2^ = 0.456). The *F* coefficients were significant in all 15 regression models (with *p* < 0.01); therefore, all models were effective in prediction. However, model 15 had the highest *F* value (*F* = 13.75, *p* < 0.01) (**Table 4**).

**Table 4:** Analysis of variance for regression model 15 showing the influence of independent variables on the dependent variable at baseline.

Model		Sum of squares	Degree of freedom	Mean of squares	F	p
15	Regression	19439.207	5	3887.841	13.753	.000^n^
	Residual	23180.748	82	282.692		
	Total	42619.955	87			

We chose model 15 as the most appropriate (**Table 5**). Model 15 explained 42.3% of the variance in QoL (adjusted *R*^2^ = 0.423), with a high level of overall effect.

**Table 5:** Model 15.

Model	R	R square	Adjusted R square
15	.675^o^	.456	.423

**Table 6** presents the results obtained from model 15, showing each independent variable’s standardized and unstandardized coefficients, standard errors, *t*-test results, and zero-order, partial, and semi-partial correlations.

**Table 6:** Regression coefficients for model 15 predicting the quality of life at baseline.

Model 15	Unstandardized coefficients	Standardized coefficients		t	p	Correlations			
	B	Std. Error	Beta			Zero-order	Partial	Part	
(Constant)	45.93	8.17		5.62	.000			
PANSS positive	.46	.18	.211	2.46	.016	.095	.262	.201
Self-care capacity	17.42	4.16	.375	4.19	.000	.530	.420	.341
Social support	9.80	4.00	.211	2.45	.016	.384	.261	.200
Financial planning	7.87	4.56	.167	1.73	.088	.385	.187	.140
AP	-15.92	5.88	-.248	-2.70	.008	-.370	-.286	-.220

In the regression model, self-care capacity (*β* = 0.375, *p* = 0.000), the need for an antipsychotic (*β* = -0.248, *p* = 0.008), the positive score of the PANSS (*β* = 0.211, *p* = 0.016), social support (*β* = 0.211, *p* = 0.016), and financial planning capacity (*β* = 0.167, *p* = 0.088) were significant predictors of QoL at the baseline.

Interestingly enough, the negative coefficient of the need for an antipsychotic suggested an inverse relationship, whereby a higher likelihood of needing an antipsychotic predicted a lower QoL. The other positive coefficients suggest the importance of these social factors in maintaining higher QoL.

Another assumption for multiple regression was that the residuals were normally distributed(**Supplementary Figure 1**). **Supplementary Figure 1** verified the normality of the distribution of the standardized residuals by comparing them with the deviations from the normal curve. We found that the condition of normality of the distribution of the residuals was met.

**Supplementary Figure 2** presents the normal P–P plot of regression standardized residuals, showing that the residuals closely followed the diagonal line, indicating that the assumption of normality was met.

### 3.4 Regression analysis at the 6-month follow-up

The other research question that our study addressed was which factors influence QoL at the 6-month follow-up. To this end, we conducted a multiple regression analysis using the same algorithm used for initial testing, in which the dependent variable was the WHOQoL score at the 6-month follow-up and the independent variables were all of the assessed factors.

We again chose the backward analysis, and 14 models were generated. We relied on the adjusted *R*^2^ value, which indicated the relevance of the model. The highest adjusted *R*^2^ value was 0.696 in model 10. The *F* coefficients were significant in all of the 14 regression models (with *p* < 0.01); therefore, all models were effective in prediction. However, model 14 achieved the highest *F* value (*F* = 27.45, *p* < 0.01) (**Table 7**).

**Table 7:** Analysis of variance for regression model 14 showing the influence of independent variables on the dependent variable at follow-up.

Model		Sum of squares	Degree of freedom	Mean of squares	F	p
14	Regression	39825.736	7	5689.391	27.450	.000^n^
	Residual	16581.344	80	207.264		
	Total	56407.080	87			

We chose model 14 as the most appropriate (**Table 8**). Model 14 explained 68.0% of the variance in QoL (adjusted *R*^2^ = 0.680), with a high level of overall effect.

**Table 8:** Model 14.

Model	R	R square	Adjusted R square
14	.840^n^	.706	.680

**Table 9** presents the results obtained with model 14, showing each independent variable’s standardized and unstandardized coefficients, standard errors, *t*-test results, and zero-order, partial, and semi-partial correlations.

**Table 9:** Regression coefficients for model 14 predicting the quality of life at follow-up.

Model 14	Unstandardized coefficients	Standardized coefficients		t	p	Correlations		
	B	Std. Error	Beta			Zero-order	Partial	Part
(Constant)	115.72	9.31		12.43	.000			
PANSS negative	-.60	.25	-.233	-2.41	.018	-.657	-.261	-.146
PANSS general	-.39	.12	-.303	-3.18	.002	-.605	-.335	-.193
Self-Care capacity	24.24	4.86	.329	4.99	.000	.553	.487	.302
Presence of medico-legal complications	-13.06	5.05	-.164	-2.58	.012	-.205	-.278	-.157
AD	-11.89	3.32	-.235	-3.59	.001	-.246	-.372	-.217
Anti-craving medication	7.40	3.24	.146	2.29	.025	.284	.248	.139
Level of education	3.93	1.45	.172	2.72	.008	.322	.291	.165

In the regression model, self-care capacity (*β* = 0.329, *p* = 0.000), the need for an antidepressant (*β* = -0.235, *p* = 0.001), the score of the general scale of PANSS (*β* = -0.303, *p* = 0.002), level of education (*β* = 0.172, *p* = 0.008), presence of medico-legal complications (*β* = -0.164, *p* = 0.012), the score on the negative scale of PANSS (*β* = -0.233, *p* = 0.018), and the need for anti-craving medication (*β* = 0.146, *p* = 0.025) were significant predictors of QoL at the 6-month follow-up.

Interestingly, the negative coefficients that decreased QoL were as follows: the scores of the general and negative scales of PANSS, presence of medico-legal complications, and need for an antidepressant. The other positive coefficient suggests the importance of the personal factor—level of education—in maintaining higher QoL. In addition, we found that anti-craving medication use increased the QoL.

Another assumption for multiple regression was that the residuals were normally distributed (**Supplementary Figure 3**). **Supplementary Figure 3** verified the normality of the distribution of the standardized residuals by comparing them with deviations from the normal curve. We observe that the condition of normality of the distribution of the residuals was met.

**Supplementary Figure 4** presents the normal P–P plot of regression standardized residuals, which showed that the residuals closely followed the diagonal line, indicating that the assumption of normality was met.

**Figure 3:**
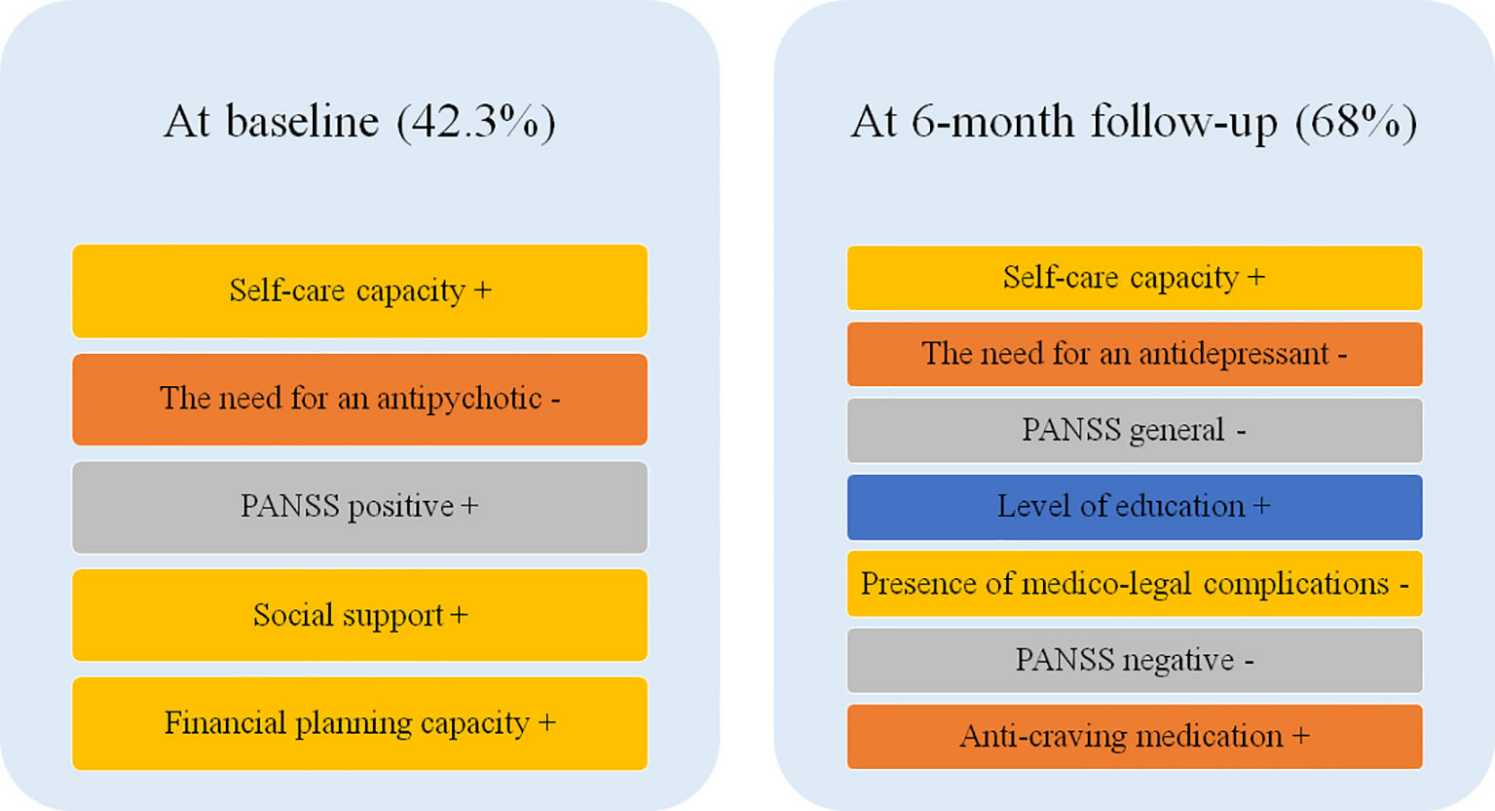
Comparison of factors influencing the quality of life between baseline and the 6-month follow-up.

In **Figure 3**, we present a comparison between the main findings of the two regression analyses, representing the different factors that influence (positively represented by “+” and negatively represented by “-”) QoL at baseline and the 6-month follow-up.

## 4 Discussion

In this study, we prospectively followed male patients with comorbid schizophrenia and AUD over 6 months. We examined multiple factors influencing the QoL and observed how these parameters differed between the two assessment timepoints: baseline and 6-month follow-up. The examined factors included AUD severity (measured using the MAST scale), schizophrenia severity (measured using the PANSS scale), social factors, personal factors, hospitalization factors, and treatment factors (categorizing patients based on their need for an antidepressant, antipsychotic, or anti-craving medication).

The study adopted an observational, prospective design, with a 6-month follow-up period. We examined 88 male patients who received individualized pharmacological treatment according to their specific clinical needs at the time of enrollment, which was considered the baseline timepoint. Treatment requirements (whether they needed antidepressant, antipsychotic, or anti-craving medication or a combination of these) were determined by the attending psychiatrist, who assessed each patient at both the baseline and follow-up timepoints. In this study, we essentially monitored these patients and their clinical evolution, seeking associations between clinical symptoms (of schizophrenia and alcoholism), personal and social factors, factors related to hospitalization (previous hospitalizations and type of admission), and the treatment regimens required.

From a prospective viewpoint, the first important result of our study was the high rate of prediction both at baseline (with a prediction rate of almost 43%) and the 6-month follow-up (with a prediction rate of 68%). This high predictive rate highlights the importance of the multifactorial approach employed in our study, in which we evaluated social and personal factors, hospitalization-related factors, clinical features of both diagnoses, and treatment-related factors. Thus, we highlight the importance of conducting an individualized and multidimensional assessment of each patient, reflecting the inherent complexity of the human psychic condition.

Second, the results of the regression analyses at baseline and follow-up must be emphasized. At baseline, the factors that predicted QoL in order of their prediction rate were as follows: self-care capacity (positive association), the need for an antipsychotic (negative association), PANSS positive score (positive association), social support (positive association), and financial planning capacity (positive association). Interestingly, we found that the score on the positive subscale of PANSS was positively associated with higher QoL, while the need for an antipsychotic was negatively associated with lower QoL. This result confirms the view that patients with dual diagnosis, who have more positive schizophrenia symptoms, have a better response when their treatment includes an antipsychotic, and this combination is reflected by the QoL of these patients. Another important aspect, which may be counterintuitive, arose from the following finding: at baseline, we encountered patients with schizophrenia and AUD who had already recovered from acute psychosis caused by AUD (because we evaluated them 7 to 12 days after withdrawal) and were in a state of alcohol withdrawal or post-withdrawal syndrome. During this period, the patients usually present with weakness, asthenia, and physical malaise, which are symptoms that may decrease the QoL. However, our patients had increased QoL and felt better, potentially owing to the residual, but not acute, positive symptoms of schizophrenia. This result may explain the finding regarding the scores on the PANSS positive scale; specifically, they may not have reflected the severity of schizophrenia to a large extent but instead may have been indicative of the recovery from the AUD crisis. In addition, based on the regression analysis, we also uncovered the social factors that increase the QoL of patients with the dual diagnosis: self-care capacity, social support, and financial planning capacity.

On the other hand, at the 6-month follow-up, the factors that predicted QoL in order of their prediction rate were as follows: self-care capacity (positive association), the need for an antidepressant (negative association), the score on the general subscale of the PANSS (negative association), level of education (positive association), presence of medico-legal complications (negative), the score on the negative subscale of the PANSS (negative association), and the need for an anti-craving medication (positive association). Interestingly, we found that the scores on the negative and general subscales of the PANSS were negatively associated with QoL, while the need for an antidepressant was negatively associated with a lower QoL. This result confirms the view that negative symptoms in dual diagnosis patients are associated with a lower QoL and, from a treatment point of view, these patients would benefit more if they were treated with an antidepressant. Another important result of the regression analysis was that it highlighted the social factors that influence the QoL of these patients: self-care capacity (if higher, higher QoL) and presence of medico-legal complications (if present, lower QoL). Furthermore, the regression analysis highlighted the positive predictive value of the level of education for QoL in these patients—a result that confirmed our hypothesis that certain individual factors may act as protective factors. Moreover, the positive predictive value of anti-craving medication use in these dual diagnosis patients highlights the improvements in QoL that this medication may produce in these patients.

Third, another interesting result was that alcoholism severity (measured using the MAST) did not predict QoL neither at baseline nor at the 6-month follow-up. This result can be explained by the fact that the MAST may not be appropriate for use in patients with dual diagnosis. Therefore, we highlight the importance of developing a validated instrument for assessing AUD in patients with dual diagnosis. Another explanation may be that in patients with schizophrenia and AUD, QoL is primarily impaired by the effects of schizophrenia rather than by those of AUD. We suggest that the psychiatric disability in these patients is predominantly attributable to schizophrenia, with AUD representing an associated condition. Nonetheless, this comorbidity requires targeted treatment to improve the QoL, a point further supported by the positive predictive value of the variable related to anti-craving therapy.

Our results align with and expand those in the growing literature on dual diagnosis populations and QoL—for example, a Turkish study found that schizophrenia patients with SUDs had significantly lower psychological domain QoL scores than those without SUDs, even though PANSS scores did not differ between groups (31). Moreover, studies of hazardous alcohol use among patients with schizophrenia found that alcohol use correlates with poorer functioning and QoL (32).

The literature on dual diagnosis more broadly emphasizes that co-occurring SUDs in patients with SMI lead to more severe symptoms, greater hospitalization periods, and worse overall outcomes (33, 34).

The role of functional capacity and social/educational resources has been documented in schizophrenia populations; for instance, a recent Bangladeshi study found that medication adherence, social support, and substance use were significantly associated with WHOQoL–BREF domain scores (35). Studies using the WHOQoL–BREF in SUD populations likewise emphasized the importance of environmental and social dimensions of QoL and effects of social support (36, 37).

Importantly, our finding that anti-craving medication use was associated with higher QoL suggests that integrating addiction treatment with psychiatric care can enhance QoL. While there is limited literature that directly reports on QoL improvement brought about by anti-craving medication in patients with comorbid schizophrenia and alcoholism, our results support integrated treatment approaches that are recommended across dual diagnosis research—for example, a research landscape analysis revealed that dual diagnosis is characterized by complex treatment needs and that integrated care is essential to improve the outcomes (33). Parallel trials in general AUD suggest that the effects of topiramate can match or exceed those of naltrexone in improving some drinking-related outcomes, highlighting the importance of medication choice and tolerability in patients with dual diagnosis; however, direct head-to-head studies in patients with psychosis are needed to clarify the relative effects (38).

Social support emerged as a significant predictor of QoL, with patients who reported receiving support showing better QoL outcomes, consistent with meta-analytic evidence highlighting the importance of psychosocial factors, such as employment, social functioning, and education about schizophrenia (35, 39).

One interesting and somewhat unexpected result was that antidepressant medication use was associated with lower QoL. The finding that the need for antidepressant prescription was associated with lower QoL may be indirectly explained by the symptom profile of patients with the dual diagnosis. These patients were primarily individuals with schizophrenia who exhibited both positive psychotic symptoms and, importantly, negative symptoms. The greater the burden of negative symptoms, the poorer the QoL and the higher the likelihood of benefiting from adjunctive antidepressant treatment tended to be. Thus, the need for antidepressant therapy should not be interpreted as a direct determinant of reduced QoL but rather as an indicator that these patients presented with more pronounced negative symptoms, reflecting a more severe underlying illness. Based on our data, the attending psychiatrist prescribed antidepressant medication for patients with more severe affective burden and who were experiencing profound disturbances, such as anhedonia, apathy, chronic anxiety, and depression, which may have been part of the negative symptoms, an independent depressive episode, or a consequence of alcohol abuse. Thus, our finding revealed that the use of antidepressant therapy was an attempt to treat a more disadvantaged subgroup of dual diagnosis patients whose potential for well-being and QoL restoration was lower. The observed association between antidepressant medication use and lower QoL should be interpreted with caution and should not be considered evidence of a negative treatment effect. It is more likely to reflect greater baseline illness severity or clinical complexity (i.e., confounding by indication), including factors such as the duration of illness, medical and psychiatric comorbidities, and medication adherence. Another possible explanation may be the presence of side effects of antidepressant therapy and how these side effects may affect QoL, even though we did not evaluate them. Literature on schizophrenia shows that while symptom control is important, subjective QoL may not always directly correlate with medication dose or polypharmacy; sometimes, just the presence of adverse effects reduces the QoL (40). This result highlights the complex balance between the clinical management of symptoms and subjective QoL, especially in dual diagnosis patients in whom there is an interplay among side effect burden, cognitive impairment, and social functioning.

Previous studies using the WHOQoL–BREF and similar tools in psychiatric and substance-using populations consistently showed that QoL is lower in populations with comorbidities than in those with either psychiatric disorders or SUDs alone—for example, Akvardar found that patients with schizophrenia and alcohol dependence scored lower on the social and psychological domains than healthy individuals (31, 41). More recently, a 2023 cross-sectional study of AUD patients in India reported mean WHOQoL–BREF domain scores of approximately 60–68 in the physical, psychological, social, and environmental domains, with demographic variables, such as marital status and years of drinking, influencing the QoL (42).

Our study extends these previous findings by focusing on dual diagnosis patients (schizophrenia and alcoholism) and by evaluating multifactor predictors, including schizophrenia-related factors (measured using the PANSS), AUD-related factors (measured using the MAST), social and personal factors, and hospitalization and treatment factors.

Our study argues for integrated psychiatric and addiction care. The literature regarding this view is split, with several research papers suggesting the superiority of the effects of integrated dual diagnosis treatments on multiple domains, including QoL, while comparative effectiveness studies have found no clear advantage of the effects of dual diagnosis treatment over those of non-integrated treatment regimens on both substance use and schizophrenia. These findings indicate that integration by itself is unlikely to guarantee positive outcomes, with the effects likely moderated by program quality, treatment intensity, and individual patient characteristics (43–46).

Several limitations of our study should be noted. First, the observational design introduces important concerns regarding confounding and selection bias. Treatment allocation was not randomized but was based on the attending psychiatrist’s clinical judgment, raising the possibility of confounding by indication. The characteristics of patients receiving naltrexone may have differed systematically from those of patients who were not receiving this medication; specifically, patients receiving naltrexone potentially had higher motivation, better treatment adherence, or less severe illness. These factors may have contributed to the observed differences in QoL. Therefore, our findings should be interpreted as associative rather than causal. Future studies using randomized controlled designs or advanced statistical approaches (e.g., propensity score matching) are needed to better address these issues.

Second, the study involved a single-center sample of 88 male inpatients, which limits the generalizability of results to broader or mixed-gender populations. The sample limits were the result of the rigorous inclusion criteria. The sample included the total number of patients who met the inclusion criteria during the study period approved at the hospital level. Initially, the study was approved for a single clinical department and a single doctor to manage patients with the comorbidity in order to standardize the clinical judgment. The decision to limit the study to a single department was made based on financial reasons related to the stock and cost of naltrexone in the hospital. The hospital preferred conducting a pilot study to determine if its results could establish whether or not naltrexone should be introduced into the standard hospital treatment protocol for patients with this dual diagnosis.

In clinical pavilions intended for the hospitalization of women, the prevalence of the comorbidity of schizophrenia and alcoholism is significantly lower (the comorbidity of depression and alcoholism is usually observed in women). Accordingly, in our country, this dual pattern of schizophrenia and AUD diagnoses is more common in the male population. Substance abuse is more common in men than in women with schizophrenia or first-episode psychosis (47). Additionally, men with schizophrenia tend to have a higher rate of comorbid alcohol/substance abuse than women (48). The prevalence of alcohol use is lower among women with schizophrenia than among men with the disease, and the dual diagnosis burden is substantial and often under-recognized in women (49). Furthermore, men have a significantly higher incidence of substance use and overall psychiatric comorbidity than women (50). Therefore, the results of our study cannot be extrapolated to women, outpatient populations, or other cultural and healthcare contexts. Our findings should be considered context-specific. Future multicenter studies including mixed-gender and more diverse populations are necessary to enhance the external validity.

Third, the study included patients who were admitted with AUD complications (such as delirium tremens), which were tested 7–12 days post-admission after clinical stabilization, an aspect that may have affected the responses compared to those in patients who were admitted without such complications.

Fourth, social factors were assessed in a relatively rudimentary manner by using simple dichotomous (yes/no) responses (for example, the presence of social support was recorded only as present or absent without recording further details regarding whether support was provided by a spouse, a partner, family, or friends, which may have limited the complexity of the social support dimension).

Fifth, some of the instruments were self-report instruments (e.g., MAST, WHOQOL–BREF), and even if a specialist may have assisted the patients in completing the assessment, the patients’ answers may have been affected by social desirability bias, recall inaccuracies, or limited insight typical of schizophrenia. Additionally, the non-significant association between alcohol severity and QoL may be partly attributable to the use of a lifetime-oriented screening instrument (MAST). Future studies may benefit from using instruments that more directly assess the severity of alcoholism or drinking patterns, such as the Alcohol Use Disorders Identification Test (AUDIT), Alcohol Dependence Scale (ADS), or Severity of Alcohol Dependence Questionnaire (SADQ).

Sixth, while regression models identified significant predictors of QoL, the results revealed association relationships rather than causal relationships.

Lastly, the research design included a 6-month follow-up period; therefore, we were not able to capture long-term fluctuations in alcohol consumption, relapse, or functional recovery that characterize dual diagnosis trajectories.

In conclusion, the present findings both align with and extend the findings in the existing literature on QoL in individuals with schizophrenia and comorbid AUD.

In line with previous research, we observed that social and functional factors, particularly self-care capacity, social support, and educational level, were among the strongest predictors of QoL. Our results reinforce the view that recovery-oriented outcomes in schizophrenia are driven not only by symptom reduction but also by social reintegration and functional autonomy. Consistent with findings reported in existing literature, negative and general psychopathological symptoms were associated with poorer QoL at follow-up. This finding indicates that persistent negative symptoms and global psychopathology exert a sustained detrimental effect on patients’ perceived well-being over time. Another notable finding was the absence of a significant association between alcohol use severity, as measured by the MAST, and QoL at either baseline or follow-up. A plausible explanation lies in the characteristics of the MAST itself, which primarily captures lifetime alcohol-related problems rather than current consumption patterns or recent drinking behavior. The finding that anti-craving medication use was positively associated with QoL at follow-up aligns with emerging evidence supporting the benefit of pharmacological interventions for AUD in individuals with a severe mental illness. Although randomized evidence remains limited in dual diagnosis populations, observational and clinical studies suggest that effective reduction of alcohol craving and consumption can indirectly improve functioning, stability, and perceived well-being. At the same time, this association should be interpreted cautiously, as treatment allocation in the present study was non-randomized. Patients who received anti-craving medication may have differed systematically from those who did not in terms of motivation, treatment adherence, or clinical stability—factors that themselves could contribute to better outcomes.

Our research suggests several practical applications:

- Integrated treatment programs for schizophrenia should systematically screen for AUD and address alcohol dependence concurrently with schizophrenia symptoms to improve the QoL.
- QoL monitoring should be integrated into the routine assessment of patients with dual diagnosis, and QoL should be considered an essential indicator of disease remission in this population.
- Enhancing functional abilities, self-care abilities, and educational or rehabilitative efforts may lead to substantial benefits in QoL beyond symptom reduction alone.
- Social support interventions (such as family involvement, vocational support, peer networks) may improve QoL in dual diagnosis populations.
- Medication regimens should be planned considering QoL and individual patient-based treatment while avoiding unnecessary polypharmacy.

## Data Availability

The original contributions presented in the study are included in the article/**Supplementary Material**. Further inquiries can be directed to the corresponding author.

## Supplementary material

Supplementary Figure 1 Residue distribution.

Supplementary Figure 2 Normal P-P plot of regression standardized residuals.

Supplementary Figure 3 Residue distribution.

Supplementary Figure 4 Normal P-P plot of regression standardized residuals.
