# Supplementary material for: Romanian male patients with the dual diagnosis of schizophrenia and alcohol use disorder: a prospective study of clinical, social, and treatment-related factors affecting quality of life
Source: Front Psychiatry. 2026 May 13;17:1780813. doi: 10.3389/fpsyt.2026.1780813 (PMC13212323; doi:10.3389/fpsyt.2026.1780813)
Supplement: Supplementary Figure 1 — Residue distribution. [file DataSheet1.docx]

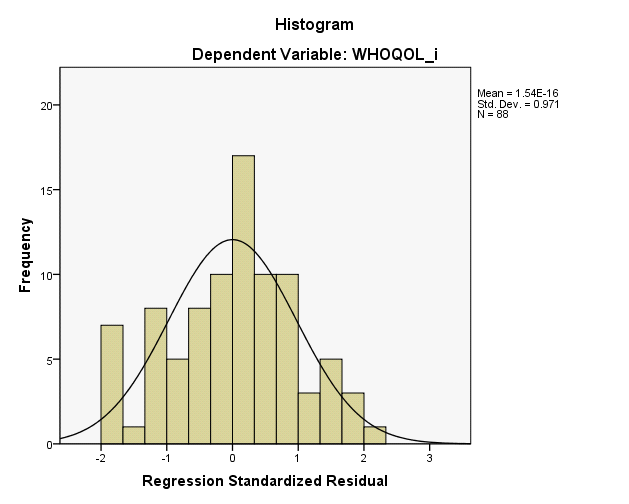


**Figure S1.** Residue distribution


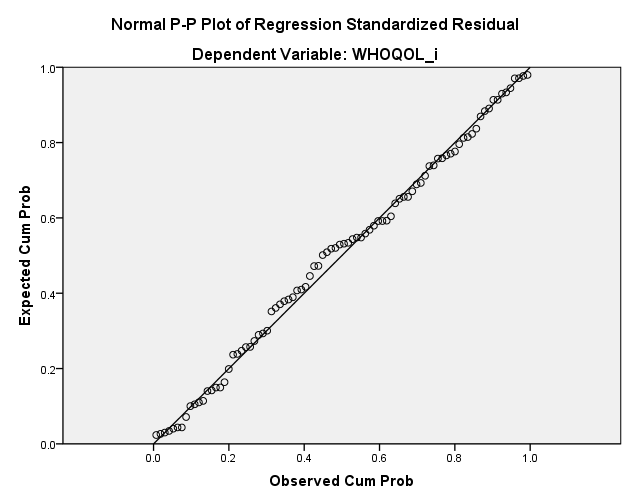


**Figure S2.** Normal P–P Plot of Regression Standardized Residuals


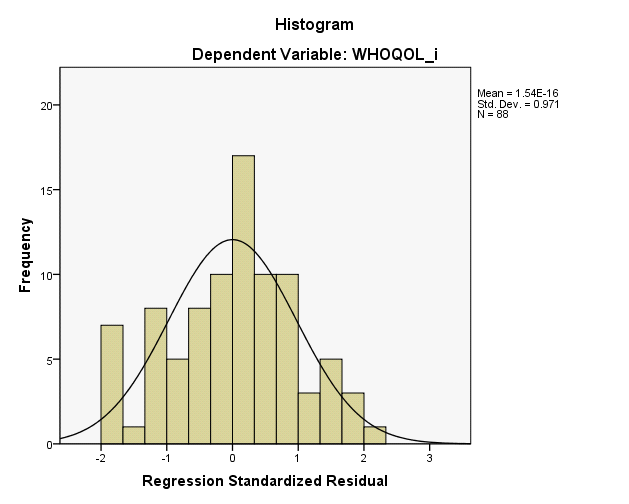


**Figure S3.** Residue distribution


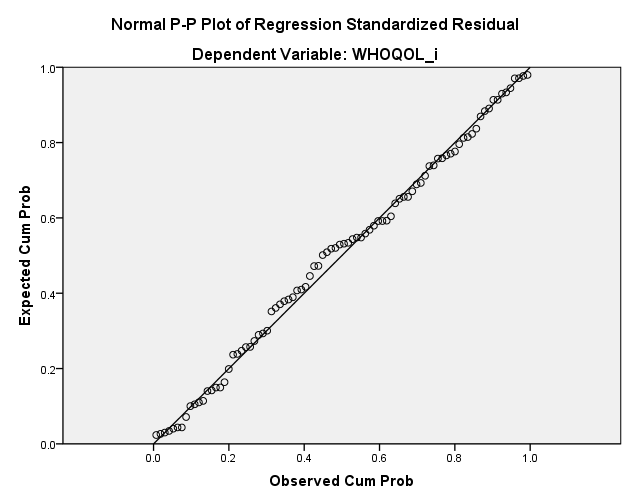


**Figure S4.** Normal P–P Plot of Regression Standardized Residuals
